# Embodied Resistance to Persuasion in Advertising

**DOI:** 10.3389/fpsyg.2016.01202

**Published:** 2016-08-15

**Authors:** Peter Lewinski, Marieke L. Fransen, Ed S. Tan

**Affiliations:** ^1^Amsterdam School of Communication Research, Department of Communication Science, University of Amsterdam, AmsterdamNetherlands; ^2^Kozminski University, WarsawPoland; ^3^Department of Media, Cognition and Communication, University of Copenhagen, CopenhagenDenmark

**Keywords:** resistance to persuasion, embodied emotion regulation, facial expression, consumer behavior, enjoyable advertisements

## Abstract

From the literature on resistance to persuasion in advertising, much is known about how people can resist advertising by adopting resistance strategies, such as avoidance, counter-arguing, and selective attention (e.g., Fransen et al., 2015b). However, the role of emotion regulation and bodily expression in resisting persuasion is so far underexplored. This is a surprising observation if one considers that at least 40% of advertisements use positive emotions (i.e., happiness) to persuade people to like the ad, brand, and product (Weinberger et al., 1995). In this article we present a framework in which we apply previous knowledge and theories on emotion regulation and embodiment to the process of resistance to persuasion. In doing so, we specifically address the role of facial expression in the course of resistance. The literature and findings from our own research lead us to propose that people can resist persuasion by controlling their facial expression of emotion when exposed to an advertisement. Controlling the expression of emotions elicited by an ad (for example refusing to smile) might be a fruitful way to resist the ad’s persuasive potential. Moreover, we argue that co-viewers can affect embodied resistance to persuasion. Showing the viability of embodied resistance to persuasion is relevant in view of the fact that ads trying to persuade us by addressing our positive emotions are ubiquitous. Embodied resistance might help people to cope with these induced positive emotions in order to resist advertisements and might therefore work as a novel and effective strategy to resist persuasion.

## Introduction and Overview

When we think about persuasion in a consumer context, we often think about advertising. This is a justified association because advertising is a powerful and lucrative persuasion tool with revenues forecasted to grow to $536 billion worldwide in 2015 (Magna Global, 2014). Advertisements often serve to increase consumption without consideration given to the needs of prospective buyers. Therefore, many observers have raised significant concerns about the potentially undesirable effects of advertising and the persuasive tactics that marketers use to engage audiences (Boush et al., 1994; Calfee and Ringold, 1994; Darke and Ritchie, 2007). People sometimes believe that brands provide them with dishonest or distorted messages in their advertisements and feel inclined to stand up to these practices. They thus seem to desire control over whether or not they are persuaded by media and advertising. Although people may know that marketers create ads designed to influence their behavior (Friestad and Wright, 1994; Wright et al., 2005), some persuasion tactics nevertheless pose a distinct challenge. Therefore, some may want more tools in their repertoire to resist these types of advertisements.

The present paper is inspired by the apparent inequity between research on the effectiveness of advertisements on the one hand, and the limited amount of research on the tools to resist them on the other. From the perspective of emotion, many strategies that people use to resist persuasion are quite broad. Marketers, meanwhile, deftly play on specific emotions in their ads. Therefore, aiming resistance at emotions that are often used in advertising is important. In other words, a productive line of inquiry might lead to ways of helping people to cope with (positive) emotions used to elicit behaviors beneficial to marketers. We therefore endeavor to uncover concrete tactics for dealing with specific emotions like happiness in the service of resisting persuasive advertisements.

In the following sections, we begin with a summary of the usage and effects of enjoyable advertisements as a persuasive tool, followed by a discussion of resistance toward persuasion. Next, we present our propositions regarding embodied resistance to persuasion grounded in the specific context of advertising and we provide first empirical support for these from studies conducted in our own lab. Finally, we discuss the framework’s implication for the theory and practice of persuasive communication and its relation to existing models.

## Enjoyable Advertisements

The use of enjoyable advertisements is omnipresent; about 40% of all advertising is intended to be enjoyable and humorous (Weinberger et al., 1995). Amusing advertisements can be defined as “all forms of smile-inducing stimuli” that are persuasive and designed to induce attitude change (Duncan, 1979, p. 302). Amusement in advertisements has proven highly effective in shaping people’s attitudes toward promoted products. Eisend’s (2011) meta-analytic test of humor in advertising credits affect with a more primary role than cognition. Eisend specifically proposes that humor enhances “hot” cognitions in general (where positive cognition outweighs negative cognition) and reduces negative cognitions through distraction effects. Humor may also evoke generic positive responses (Gulas and Weinberger, 2006) and lead to transfer of affect through evaluative conditioning (De Houwer et al., 2001). By employing humor, amusing advertisements enhance many of the typically studied outcome variables in advertising research. Eisend (2009) concluded in his overview and meta-analysis of the effects of humor in advertising that the use of humor, in general, enhances attitudes toward the ad, positive affect, attention, cognitive positive responses, and recognition. Since positive emotions and humor are used extensively in advertising and often result in positive effects from the perspective of marketers, it is pertinent to focus on diminishing the effects of amusement and humor when motivated to resist persuasion.

## Resistance to Persuasion

We define resistance to persuasion as a usually motivated attempt to withstand persuasive attacks, conforming to the use of the concept by other researchers (e.g., Zuwerink and Cameron, 2003). Much is known from the literature regarding the strategies people employ to resist persuasion. Fransen et al. (2015b) recently proposed three types of resistance – avoidance, contesting, and empowering – to categorize the strategies adopted by people to resist the effects of advertising. Avoidance strategies consist of avoiding the ad altogether or only paying attention to those parts of a message that confirm existing beliefs. Contesting strategies entail a set of approaches that actively counter specific elements of the ad (e.g., counter arguing, source derogation, message derogation), while empowerment strategies focus on confirming existing attitudes (e.g., attitude bolstering and social validation). Fransen et al. (2015a) have then latter added one more type, biased processing, which refers to processing a message in such way that it fits existing attitudes and behavior or reduces it’s relevance. Further, Kirmani and Campbell (2004) and Knowles and Linn (2004) proposed their own frameworks of resistance to persuasion, which we will not review here as we chose to present the most recent conceptualizations, i.e., Fransen et al. (2015a,b).

What is relevant for our reasoning here is that none of these frameworks and proposed strategies deals specifically with resisting the positive *emotional* or *affective* responses that are often elicited by advertisements and have been found to increase persuasion. Because the use of positive emotions in ads is ubiquitous and effective, the study of how consumers can resist persuasion by focusing on the emotions in the ad is clearly worthwhile.

### Emotions and Embodiment in Regulation for Resistance

Although researchers know that marketers try to persuade people by using positive emotions, there is insufficient knowledge on how to resist these kinds of ads. Companies use amusing ads to influence us, and more to the point, to make us smile with enjoyment, which may subsequently results in persuasion. On this account, we propose that consumers might protect themselves against advertising’s effects by “resisting” specific emotions that ads induce as part of their persuasive potential. They might accomplish this resistance by regulating their emotions when exposed to an ad (e.g., not smiling) away from the effects targeted by the ad. The literature supports the assumption that emotion regulation may find a viable starting point in the bodily expression of emotion, as we will argue below.

In a special issue on embodiment, Krishna and Schwarz (2014) presented recent findings demonstrating that people’s attitudes and intentions are often embodied, at least partially. The implication is that the body plays a role in making up people’s minds. Bodily states or changes in bodily states serve as sources of information when forming attitudes and intentions (Herbert and Pollatos, 2012). In the context of advertising, embodiment means that people smile at an amusing ad and interpret the related feelings of happiness as information to evaluate the ad and the promoted product. In other words, smiling at an ad causes us to like it more. When seeking to resist ads of this type, self-regulating this almost reflexive smiling response might ultimately reduce liking for the ad. We propose that people can resist advertising effects through modulating their “default” bodily response to the ad, including emotions targeted by the ad and the ensuing attitudes. We will refer to this process as embodied resistance to persuasion.

Moreover, the literature on persuasion and resistance reveals an emphasis on the individual consumer at the expense of other perspectives, such as group effects. Ads, like most forms of television programming, are often consumed in company, e.g., at home with family relatives, or friends. Ads that attempt to persuade using emotion or humor may derive added effect from emotion sharing in co-viewing persons. Consequently, a reasonable proposition might be that resistance to persuasion can likewise derive benefit from sharing emotion regulation across co-viewing individuals. We propose more specifically that mimicking emotional expressions of other viewers is key to shared emotion and shared regulation of emotion. Shared emotion regulation may be relatively easy because the individual viewer of an ad is “supported” by a co-viewer. Expanding the unit of analysis from the individual to the group, we speculate that under some conditions shared emotion regulation transforms into joint resistance to persuasion. In sum, we suggest embodied emotion regulation as a viable, new strategy for resistance to persuasion that can also be shared among co-viewing consumers.

## Embodied Resistance to Persuasion

**FIGURE 1:**
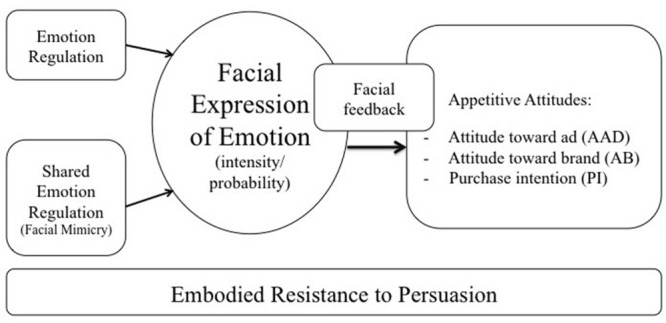
**Embodied resistance to persuasion**.

How does resisting enjoyable advertisements by suppressing facial expression of happiness function in detail? We explain this in more detail in the theoretical framework below (summarized visually in **Figure 1**). We introduce each of the components and state the main tenets of embodied resistance in the subsequent sections.

### Emotion

Embodied resistance to persuasion is a particular form of emotion regulation, namely regulation in the service of resistance. We first must define what we mean by emotion, facial expression, appetitive attitudes, and emotion regulation, mainly by singling out the definitions used from alternative theoretical approaches. Research on emotion has been dominated by Ekman’s (1972) Basic Emotion Theory. New evidence (e.g., see Russell et al., 2003; Barrett et al., 2011; Lewinski, 2015c), however, has challenged this well-established theory, especially its claim that the commonly understood emotions are each discrete phenomena, category bounded, easy to produce, and readily recognizable (Russell, 2003; Barrett, 2006). We chose the componential conceptualization of emotion as an alternative that, like the basic emotion view, permits labeling emotions and expressions as categories, but unlike Basic Emotion Theory does not restrict the categorization of any emotion to a set of basic labels. On the contrary, emotions can be labeled according to outcomes of an ongoing process of *appraisal* of the emotional stimulus, giving rise to an essentially open set of labels. Obviously, any traditional basic emotion labels are not excluded from these outcomes.

Our framework has its starting point in Scherer’s (2004, 2009) componential process model of emotions which conceives emotions as synchronized reactions of modules that operate in interdependency (Scherer, 2001). As with all emotion theories, affect is the foundation of emotion. Emotion is thus comparable to a luxury variant of affect: it contributes to affect a number of component emotion processes notably (1) an elaborate appraisal of the stimulus, including feeling and action tendencies as component processes, and (2) preparation for action. Scherer’s model conforms to the appraisal-action schema observed in all major emotion theories. However, in our use of the componential model, the importance of the action component is especially pronounced. We subscribe to a premise not explicit in Scherer’s model, namely that action readiness is a sine qua non for emotion (Frijda, 2007). We explore these concepts in more detail below.

#### Facial Expression

Facial expressions of emotion are semi-universal sequences of facial muscle contractions associated with the emotional state of a person. The traditional view of Basic Emotion Theory considers these expressions discrete, innate, and culturally universal (e.g., Ekman, 1972; Ekman and Cordano, 2011). However, evidence concerning the relationship between emotion and facial expression subverts the notion of basic emotion categories being related to fixed expressions (e.g., Jack et al., 2012; see Fernández-Dols, 2013 for a review). Recent social and psychological constructionist approaches (e.g., Russell, 2003; Barrett, 2009; Mesquita, 2010) propose that distinct emotions do not have singular, unique manifestation in facial expression, and are likewise not considered natural kinds associated with dedicated brain circuits.

We consider in particular that the set of traditional basic emotion expressions may be part of the much larger or indeed open array of emotion–expression combinations. For instance, one may safely assume that a smiling person is in a happy mood (e.g., Reisenzein et al., 2013). However, uncertainty arises when it comes to what aspects of emotion are “expressed” through a smiling face. Following the logic of functional accounts of emotion, facial expressions have been argued to reflect forms of action readiness (Frijda, 2007). The following practical example illustrates the use of attending to functional aspects of facial expression. Imagine someone observing you smiling intensely while watching an enjoyable commercial on television. This observer cannot be sure about your feelings at that particular moment but probably infers that when you are pulling your lip corner up (i.e., smiling), it means you are feeling happy. Frijda and Tcherkassof (1997) would argue that you are not only smiling and feeling happy, but that you are also expressing favorable action tendencies to approach the commercial. Marketers and advertisers have taken note of this relationship because they are interested not only in how consumers emotionally respond to an advertisement, but probably even more so in how consumers’ feelings relate to behavioral tendencies toward the commercials, products, and brands. Following the action tendency view of emotion expression, we address the general question: can we tell from facial expression whether consumers faced with an advertisement have a particular action readiness toward the advertised product? More to the point is the question: do they want it? We subscribe to the hypothesis that facial expression of emotion reflects a state of action readiness (Frijda and Tcherkassof, 1997). In addition, we argue that action readiness (Frijda et al., 1989) in viewing ads takes the form of appetitive attitudes (as defined in Fishbein and Ajzen, 1975) toward ads, products, and brands.

### Appetitive Attitudes

Fishbein and Ajzen’s (1975), Ajzen and Fishbein (1980) definition is the starting point for our conceptualization of attitudes as action-oriented emotional responses. An attitude is the sum of the beliefs about the outcome of one’s behavior with regard to the attitude’s object. Ads, brands, and products can be objects of attitudes and emotions. Eagly and Chaiken (2007) recently defined attitudes as specific beliefs, namely evaluations of experience with the object. Attitudes represent a tendency to “evaluate a particular entity with some degree of favor or disfavor” (Eagly and Chaiken, 2007, pp. 598–599). Its property of a favorability evaluation is why attitude may be called appetitive.

Eagly and Chaiken (2007) distinguish three evaluative components in attitudes: cognition, affect, and behavior. These can be mapped onto the components of emotion. Cognition and affect evaluation correspond with appraisal of the object and global action motivation. For example, Breckler (1984) showed that affect measures correlated with physical distance taken to an object. The behavioral evaluation component of attitudes corresponds with what exactly one emotionally would like to do with the object. For instance, a readiness of approach to an ad evaluated as funny is specified as a tendency to engage in interested exploration, to pay attention, be with the ad, identify or care for the brand, possess the product and others (see Frijda, 2007). In recent years, attitudes, too, have been considered embodied inclinations, that is, attitudes involve a readiness of the body to act according to the evaluation (Niedenthal et al., 2005).

In the realm of advertising, the ad itself, the brand, and product purchase intentions are objects of appetitive attitudes. Because the model of emotion adopted here involves synchronized rather than strictly subsequent emotion component processes, we can only loosely sketch the temporal aspect of the emotional process. While viewing an amusing ad, typically a readiness to approach arises in the wake of or simultaneous with its appraisal as funny. In addition, while viewing or immediately following an ad, this approach motivation corresponds to distinct appetitive attitudes, each having their own behavioral implementations. From our embodied persuasion perspective, we propose, they include (1) immediate liking for the ad – involving a desire to attend it to the full and enjoy it; (2) brand liking – involving a tendency to identify with the brand – to associate the self with it; and (3) purchase intention – involving the tendency to put forth effort and invest resources to possess the product. Regulation of these emotions and their subsequent effects on appetitive attitudes might be crucial in persuasion and resistance processes.

### Emotion Regulation

Emotion regulation is a conscious or unconscious sequence of steps taken to control or change emotions. The process of emotion regulation starts when internal or external stimuli trigger, through a semi-stable individual threshold, the affective appraisal. The cultural- and individual-specific patterns of self-regulatory actions lead to modification of the default response and hence final behavioral outcome (Gross and Thompson, 2007). Many types of emotion regulation have been reported (e.g., see Brans et al., 2013). Reflection, rumination, and distraction are examples of emotion regulation strategies, but we focus here on the two most general ones: antecedent- and response-focused strategies (Gross, 2002).

Cognitive reappraisal is an antecedent-focused strategy. Cognitive reappraisal, as a down-regulating strategy, changes the perception of emotional events to become more objective and analytical, decreasing felt emotion. For example, someone who has recently lost a parent can think, “Dying is natural to life and it happens sooner or later.” Up-regulation leads to perceiving events in a subjective and involved manner, which increases felt emotion (Lazarus and Alfert, 1964). As a cognitive strategy, reappraisal modifies both appraisal and action readiness. Importantly, cognitive reappraisal is capable of increasing resistance to temptation. For example, Leroy et al. (2012a,b) found that reappraisal could make a task more appealing and temptation less attractive, thus aiding in resisting temptations.

Expressive regulation is the response-focused emotion regulation strategy that can either suppress or amplify outward signs of inner feelings (Gross and John, 2003). Suppression of bodily expressions has an inhibitory function and is generally associated with poorer well-being and psychological functioning (e.g., Svaldi et al., 2012). Amplification has an excitatory function, as it intensifies the organism’s physiological responses. People experience stronger emotions when activation of the sympathetic nervous system is amplified (Demaree et al., 2004). As the name implies, expressive regulation taps more directly into the expression of emotion than cognitive reappraisal does.

Returning to our case of positive emotions provoked by enjoyable ads, we propose that amusing ads typically lead to an appraisal as “funny” and attractive, related to an approach motivation. This appraisal and the ensuing action readiness might be regulated, working toward controlling one’s attitude with respect to the amusing stimuli, e.g., an ad. Assuming such self-regulation is feasible, emotion regulation is a tool that consumers could adapt for purposes of their own “defense” against persuasive advertising. From this perspective, we believe that our study of emotion regulation will contribute substantially to the literature of resistance to persuasion. Emotion may be regulated through adjusting one’s thought patterns, feelings, or expressions. From those three ways, expression regulation is crucial to embodied emotion regulation, a concept discussed below.

#### Embodied Emotion Regulation through Facial Expression

We posit that bodily expression is a privileged point of application for emotion regulation. A general argument for this assertion is that emotions as a whole (including attitudes as emotional responses) are themselves embodied. All component systems of emotion appraisal, global motivation, and responses, that is, specific action tendencies and expressions have been shown to be affected by instructions or implicit cues leading to different body postures (Niedenthal et al., 2005). Therefore, it can be expected that the control of bodily expressions can affect all other emotion components.

Next, several arguments exist in favor of specifically facial expression as a privileged nexus of emotion regulation among the bodily concomitants of emotion. Facial muscles are hypothesized to have direct, two-way connections with the experienced and expressed emotion (Friesen and Ekman, 1983). Facial expression regulates emotion strength because facial expression feeds back on felt emotion intensity (Leventhal, 1984). Facial efference theory (Zajonc, 1985; Adelmann and Zajonc, 1989; Zajonc et al., 1989) takes as a starting point the well-known fact that facial musculature is controlled by afferent input originating in the brain’s emotion centers and adds to this that, in turn, facial musculature activates efferent neural output to the emotion centers. Research in support of facial efference theory presents compelling biological evidence of facial muscle contractions influencing affective experiences by regulating blood flow (most prominently in the nasal area) to the brain.

The facial feedback hypothesis goes one-step further than postulating an efferent influence of expression on feeling. Indeed, this hypothesis is a “causal assertion that feedback from facial expression affects emotional experience and behavior” (Buck, 1980, p. 813). Strack et al. (1988) found (however, see the latest replication of Wagenmakers et al., in press), for example, that a forced smile resulted in higher humor appreciation. Zajonc (1985, p. 16) went on to demonstrate that “facial muscles act as ligatures on facial blood vessels and thereby regulate cerebral blood flow, which, in turn influences subjective feeling.” For example, Zajonc et al. (1989) asked participants to read aloud stories containing ü (U-umlaut), a vowel that involves action of the corrugator muscle and nostril constriction. Those two movements are typically part of negative emotion expression. As predicted, reading aloud “Jürgen,” “füchse,” and “hühner” lead to higher forehead temperatures in contrast to stories containing “Peter,” “hunde,” and “katzen,” because hypothetically the utterance of ü caused an airflow cooling the brain. This efferent influence presumably caused differences in experienced emotion. Participants reported liking the non ü-story more even when participant origin, content recall, or interest in German language were controlled for.

In sum, embodied resistance to persuasion could in principle deploy other means than facial expression or facial expression alone (e.g., breathing slowly or shaking the head in “no” gesture), but facial expression is an essential, arguably privileged and up to now most researched component of embodied emotion regulation. Therefore, we focus our theorizing mainly on facial expression.

#### Shared Emotion Regulation

Emotion regulation often occurs in the presence of others. We propose that a special form of embodied emotion regulation is mimicking others’ facial expression and that facial mimicry is more spontaneous than emotion regulation. Response- and antecedent-focused emotion regulation is typically private. On the other hand, people often view ads together. One way, then, that emotion regulation might be extended from the isolated individual to a social situation involving a co-viewer is through subconscious mimicking of facial expression.

Facial mimicry is the action of specific muscles’ regions in response to others’ facial expressions (Bush et al., 1989). This phenomenon occurs automatically and spontaneously whenever another person is co-present and visible. The mere perception of facial expressions of others’ emotions activates observers’ facial muscles – as measured by facial electromyography – that correspond to the perceived emotion (Dimberg, 1982; Lundqvist, 1995). Facial mimicry occurs fast – within 300 ms (Dimberg et al., 2002) – and it may even be an innate aspect of perceiving others (Niedenthal et al., 2005). Importantly, we adopt Bush et al.’s (1989, p. 32) definition of facial mimicry because it involves the process of mimicking, which modulates the “(…) subject’s own expressive displays rather than one-to-one mimicry outcomes”.

We propose that mimicry combined with facial feedback results in shared emotion. An example is that when two smiling persons mimic each other, mutual feelings of liking are enhanced (for a review, see Lakin et al., 2003). As far as the mimicked other is regulating their emotion involving facial expression, the perceiver will follow the other in the regulation. Through this process, expression of emotion and its regulation in one individual will affect co-viewers and vice versa. Thus, facial mimicry may be the vehicle for interpersonal sharing of emotion but also of interpersonal sharing of regulation.

Competent consumers may deploy shared emotion regulation. For instance, imagine that a parent’s disapproving facial expression in reaction to an ad of appealing but sugar-rich chocolate bar is mirrored by a change in the expression of a child watching the parent. Owing to properties of facial feedback, the child’s initially positive facial expression is down regulated, leading to suppression of the child’s positive feelings toward the amusing advertisement of an unhealthy but appealing chocolate bar. In line with this example, Rychlowska et al. (2014) showed that blocking mimicry made true and false smiles look the same. Thus, blocking facial mimicry (or showing opposite expression) may reduce children (or adults’) ability to accurately interpret emotional signals delivered by the ad and hence decrease the ad’s intended persuasive impact.

### In Summary

We propose that the complete process involved in embodied resistance to persuasion is the following: in the default situation of viewing a properly enjoyable ad, consumers feel a substantial degree of happiness, as typically they do not regulate their emotion in any way. The contents of the amusing advertisement are appraised as amusing, and a corresponding appetitive action readiness is automatically incited. These antecedent components cause emotional responses indicative of happiness. One is the subjective feeling of happiness, another is its expression through facial movement, and a third is appetitive attitudes – specified forms of the readiness to pleasantly engage with the ad, brand, or product. Should a consumer be inclined to resist amusing ads, response-focused emotion regulation that suppresses appetitive attitudes will apply first to the facial expression response, because conscious control of facial expression is easy in comparison to other response systems. Likewise, we argue that in the event a consumer applies antecedent regulation, facial expression will again be primarily affected for the same reason, namely that facial expression is relatively easy to hold in check. Because of the interrelatedness of emotional response systems and, perhaps more particularly, the relatedness of facial expression with the appetitive attitude response system, suppression of facial movements propagates to lower feelings of happiness and appetitive attitudes. Through a feedback mechanism, suppressed facial expressions of happiness also influence (1) antecedent components of the emotions, i.e., appraisal of the ad as funny, and (2) the emotional approach tendency toward the ad. To the degree that this mechanism persists, the consumer exhibits embodied resistance to persuasion. When the ad is part of a co-viewing experience, facial mimicry contributes to shared emotion regulation. Mimicry involves changes in the consumer’s facial movement that is targeted by the ad, propagating subsequently to appetitive attitudes through facial feedback. Mimicking facial expressions compatible with the amusing ad enhances the target consumer’s happiness and attitudes toward the ad, brand, and product, while mimicking ad-incompatible facial expressions will suppress happiness and attitudes.

### Empirical Evidence for Embodied Resistance to Persuasion

The empirical literature already consists of some research supporting the basic tenants of embodied resistance to persuasion. We review some of those studies below, which come from our own lab. We chose facial expression and emotion regulation as the focus of our studies because, as argued before, one prominent form of bodily expression is facial expression and another prominent form of self-regulation is emotion regulation. Therefore, we focus on resistance toward persuasion by facial expression manifested in emotion regulation and facial mimicry. The latter phenomenon expands the notion of embodied emotion regulation to social situations of individual consumers watching ads in the company of others. Our primary aim is thus to highlight the role of facial expression in suppressive emotion regulation for embodied resistance. The first, basic question is whether facial expression reliably predicts attitude change. The second question is whether emotion regulation of facial expression results in resistance toward persuasion. The third question is whether a shared form of emotion regulation – facial mimicry – influences facial expression and therefore attitudes of the consumers. In sum, the three testable hypotheses, we propose are:

During exposure to an enjoyable ad:

• H1: Facial expression of happiness predicts intensity of appetitive attitudes;
• H2: Suppressing facial expression of emotion helps consumers in downward regulation of appetitive attitude;
• H3: Mimicking other consumers’ incompatible expressions (incompatible-mimicry) inhibits target consumers’ facial expression of happiness and subsequently their appetitive attitudes.

In most of our studies presented below, participants were exposed to 30 s enjoyable video advertisements while their facial expression was estimated by automated facial coding software – FaceReader (for technical details and validity measures see Lewinski et al., 2014a). FaceReader’s estimation of intensity of facial responses were afterward related to consumers’ attitudes toward the ad and buying intentions. The majority of the experiments were carried out in cloud-based FaceReader Online through MTurk, which heightened the experiments’ ecological validity in comparison to standard lab experiments as participants were recorded in their own homes using their own computers as in a standard ad watching situation.

In Lewinski et al. (2014b), we demonstrated that the intensity of facial expression predicts the intensity of consumers’ appetitive attitudes in response to enjoyable advertisements. In that experiment participants viewed amusing advertisements. An emotional response to the ads was assumed to involve Frijda’s (2007) action tendencies, such as *approach* and *be with* the ad and the brand, and to *possess* displayed articles. In this study, approach and be-with tendencies were operationalized as ad liking (Phillips, 2000) and brand liking (Chattopadhyay and Basu, 1990). Participants were recorded watching three popular high, medium, and low amusing video advertisements. Facial expression during exposure to the commercials was coded using FaceReader. Ad and brand liking were measured afterward. In the high and medium, but not in the low amusement conditions, positive correlations were found between happiness scores and appetitive attitudes (i.e., attitude toward the ad and the advertised brand). No such correlations were obtained for any other basic emotion (sadness, anger, surprise, fear, disgust) in any conditions. In a similar vein, Lewinski (2015a) showed that facial expressions of speakers, coded using FaceReader, predicted the number of video views on a YouTube channel, 8 months after, controlling for baseline views. Specifically, more of facial expression of happiness and sadness (both approach tendency emotions) and less of surprise expression (ambivalent emotion as to approach tendency) correlated with higher popularity of the video (as defined by video views), an outcome variable similar to ad liking. We propose that we have found initial support for the hypothesis that facial expression not only reflects communicative intent, feeling, mood, or appraisals, but may also equally reflect appetitive attitudes.

In Lewinski et al. (2015, February), we tested consumer resistance to persuasion through embodied emotion regulation in seven facial coding experiments. Across seven experiments, we showed that response- and antecedent-focused emotion regulation decreased (increased) positive (negative) responses to a variety of advertisements. In five experiments with amusing advertisements, we demonstrated a causal mediation path from emotion regulation to expression and further down to attitude change, although we did not fully replicate the same pattern for disgusting ads. Specifically, we found that expressive suppression and cognitive reappraisal had similar inhibitory effects on facial expression of happiness, which subsequently led to lower positive attitudes about the ad, brand and intention to buy. We assume that those lowered attitudes on the side of the consumer are indicative of a successful resisted persuasion attempt. Even though, in those experiments, other resistance to persuasion strategies (e.g., source derogation) were not compared directly with emotion regulation strategies, three experiments introduced effort in emotion regulation as an explanatory variable. Those subsequent experiments showed that the effort in not feeling happiness or not feeling anything (i.e., emotion regulation) was a significant predictor of intensity of facial expressions, and hence resisting persuasion depended on it, which in turn meant that the more consumers tried to regulate their emotional experience, the better off they were (i.e., more successfully resisted the advertisement).

So far, the viewing situation was limited to the individual consumer watching an ad in isolation. In Lewinski (2015, Unpublished, June) and in Lewinski et al. (2016), we added tests of shared ERP where we demonstrated effects of facial mimicry on consumers’ attitudes and intentions in three experiments. In this study, we simulated a co-viewer using an avatar. Across three experiments, we demonstrated that during exposure to an amusing advertisement, shared emotion regulation modified consumers’ happiness and subsequently their appetitive attitudes. Specifically, we found that consumers’ incompatible mimicry – manipulated through a “disgusted” avatar – decreased consumers’ felt and expressed happiness, which in turn caused lower attitudes and intentions. In one of the experiments, we included an eyetracking manipulation check to rule out the possible confounding effect of the presence of avatar. To precisely manipulate facial mimicry in the main studies, we developed an innovative method of a virtual avatar embedded into an advertisement, which was meant to reflect participants’ anticipated facial movement pattern. We concluded that resistance to persuasion is enhanced by the presence of a skeptical co-viewer expressing their negative attitudes through facial expression.

Importantly, all support from empirical data described here came from experiments with random assignment and with a sample drawn from a representative American population (thus not a student sample). This means that any alternative predictor, such as the use of additional resistance strategies by subjects or sociodemographic status was spread equally across the conditions and hence cannot explain the findings. Further, the type of advertisement tested in the experiments, which contained different types of content, was judged as amusing and intended to evoke generic laughter responses. This is relevant in light of research on evaluative conditioning and the impact of resistance motivations as discussed by Sweldens et al. (2010) or Fransen et al. (2015a).

The studies reviewed above are a first empirical test of our framework concerning embodied resistance to persuasion. However, more empirical studies are needed in order to test the more general framework as well as the specific role of different expression systems, different types of ads and different types of attitudes (e.g., implicit attitudes).

## Implications

### Theory

We believe that our focus on emotion regulation as a resistance strategy contributes to filling the surprising lack of conceptualization on the role of emotion and embodiment in resistance to persuasion processes. As revealed in the introduction, only a handful of resistance strategies have been identified. So far, none of these strategies is emotion-specific (e.g., Knowles and Linn, 2004; Fransen et al., 2015a,b). Comprehensive theorizing modeling the dynamic, sequential, and componential nature of emotion and expression in persuasion is even more scant. In the current paper, we applied existing knowledge on embodiment and emotion regulation to the process of resistance toward persuasion. The widely known Approach–Avoidance Model of Persuasion (Knowles and Linn, 2004) includes only so-called Alpha and Omega strategies for attitude change (respectively making the message more attractive or focusing on reducing resistance to it), both of which lack an explicit emotion component. A recent paper by Fransen et al. (2015a) reporting three different types of resistance strategies (avoidance, contesting, and empowering) does not include emotion as a prominent component. To address this shortcoming in the literature on resistance to persuasion, we presented a new type of strategy that consumers may use to resist persuasion of (amusing) advertisements by regulating their own emotional and bodily responses. We explicitly revealed the mechanisms of embodied resistance to persuasion as well as its boundary conditions and specific elements.

The presented framework has further immediate implications for the theory of resistance to persuasion. For the first time, we consider the effects of a resistance strategy specifically tailored to the emotional content of an advertisement. We propose that positive attitude effects of amusing advertisements might be counteracted through different forms of cognitive reappraisal and expressive suppression, as well as incompatible facial mimicry. We furthermore argue that a model of regulation and resistance effects needs to take the expression of emotion into account.

A second contribution of the ERP framework is that it incorporates the body into consumers’ self-awareness and self-regulation. The possible underuse of embodied emotion regulation is somewhat ironic given the immediate, permanent accessibility of the resource in question. Our bodies link our internal perceptions and actions to situations and stimuli in the world. In principle, individuals have exclusive control over their bodies’ movements. The body is the primary and most-trusted source of information on external situations and internal states, and an ever-available instrument to act. In some situations, controlling the body may be easier than controlling the mind, as in the case of cognitive depletion (Wheeler et al., 2007). Competent consumers might recognize that they can to a large extent be master of their thoughts and emotions because these are embodied and situated.

#### Emotion Regulation

In our empirical studies we made a distinction between two slightly different ways of regulating emotions, one through direct instruction and another through facial mimicry as explained in the overview of experimental studies. We proposed that the two work in a similar manner but we do not exclude the possibility that there are important differences. It could be that response-focused emotion regulation (expressive modulation) influences the expression and hence attitudes both upward and downward. However, facial mimicry could have suppressive effects only. One possible explanation for this qualitative difference could be that when consumers’ expressions are already compatible, they do not feel the need to mimic one another in order to up-regulate their experience. However, that reasoning would contradict findings by Raghunathan and Corfman (2006). Another explanation would be a simple ceiling-effect, which could be tested by including more neutral or ambiguous advertisements instead of only amusing ads. In any case, this issue is left as an open question that awaits further investigation.

#### The Role of Facial Expression

Our framework assumes that appetitive attitudes are immediate outcomes of consuming the advertising stimuli. Throughout, we forwarded the proposition that facial expression predicts attitudes rather than the other way around. Hence, smiling at an advertisement means you like it more. However, situations are conceivable in which appetitive attitudes may cause increased facial expressions of happiness. Short-term appetitive attitudes could potentially develop into long-term ones, becoming *a priori beliefs* about an ad or brand resulting from repeated exposure to the ad. Consequently, any time the ad is presented, beliefs are automatically activated, instigating the appropriate facial expressions. The attitudes considered in our studies have only immediate post-viewing appetitive attitudes within their scope. Predictive effects of longer-term *a priori* beliefs on facial expression are left unaccounted for.

However, the empirical studies reviewed did throw a consistent light on the direction of causality in the case of immediate viewing effects. We were faced initially with a causal direction issue: do I smile because I like an object or, conversely, do I like it because I smile? Lewinski et al. (2015, February) tested the latter possibility thoroughly and found it held up to scrutiny. Thus, we are confident to add to the literature on facial feedback a strong case for facial expression as a causal condition for affective, immediate, and post-viewing liking responses.

##### Advantages of Measuring Facial Expressions

An often-used method to assess how consumers react upon amusing advertising is to explicitly ask them what they think about an advertisement and how they think they *feel* about it. However, asking people directly how they feel is not only cognitively demanding and difficult for the subject, that interrogative pathway also brings undesired effects into the mix, such as increased self-awareness (Pryor et al., 1977) and social-desirability (Arnold and Feldman, 1981). Moreover, as self-report measures they are incapable of capturing a number of biologically anchored emotional expressions that are inaccessible to the subject’s awareness. Physiological registrations can offer a solution to the shortcomings of explicit verbal measures of cognitive and emotional states. One such physiological measure is facial emotional expressions, which play a prominent role in our model and hence help in overcoming such disadvantages of self-reported emotions. Further automated coding of such expressions, with software such as FaceReader, eliminates the human factor in coding, which often can be less accurate, under certain conditions than coding algorithms (see Lewinski, 2015b), trained on objective material (such as in Olszanowski et al., 2015).

### Practical Implications

#### Consumer Competence

Embodied resistance to persuasion aims to increase consumer competence by empowering them with additional resistance “tools” to counteract deliberate persuasive attempts that use amusement. The insights into embodied resistance to persuasion are relevant for consumers, consumers’ interest groups and governmental and non-governmental consumer policy organizations. Consumer organizations, too, have empha sized the need for tools that help consumers to act autonomously when they are faced with consumer product supplies. The European consumer organization BEUC (Bureau Européen des Unions de Consommateurs), which represents more than four million consumers from a few dozen national consumer organizations across thirty-one European countries, has explicitly stated:

“Empowering consumers is the holy grail of current EU strategy and research. It is also a policy target for national governments, often in tandem with policies for smarter regulation or deregulation. It means that consumers take decisions and choices into their own hands where they can – provided that they have the right tools to do so. (…). If the 500 million EU consumers have all [right tools], they can influence markets with their collective power. (…). The reality, however, as our members tell us, is rather different. Numerous elements converge to disempower consumers (…). Too often companies make deliberate use of consumer information fatigue and their *behavioral biases* in their *communication strategy*” (The European Consumer Organisation [BEUC], 2012, p. 7; “EU Consumers’ 2020 Vision,” emphasis added).

In a consumer context, embodied resistance to persuasion holds promise as a tool empowering consumers and thus adding to their competence. The behavioral control that it involves adds to available elements of consumer competence such as counter-arguing or attitude bolstering. In addition, for special groups of consumers who for one reason or another cannot efficiently resist appealing messages through counter-arguing or attitude bolstering, the body may be the only easily accessible resource for resisting persuasion. This is because one prominent advantage of embodied resistance to persuasion as a strategy is that it applies to behavioral expression in the first place.

#### Disadvantaged Groups

Specifically, consumers who are not proficient in generic resistance to persuasion and have difficulty dealing with cognitive instructions well can benefit from instruction to control their bodily expressions. For example, instructing such regular consumers to feign a specific facial expression, e.g., lowering their brows could help them to resist persuasion attempts. They might interpret their bodily act of brow lowering as the physical manifestation of their own thinking, possibly enhancing cognitive performance.

Consider that a young child who cannot yet counter-argue a persuasive message is already able to inhibit his or her smiling at an appealing object. Parents could show their child how to suppress facial expressions of happiness in response to a tempting ad, whereas they could not make the child comprehend and follow a verbal instruction to counter-argue the message. Parents can guide children in resisting persuasive messages by demonstrating how to counteract persuasion using their body. This assumption stems from and would be compatible with the findings from the well-known still-face paradigm in mother infant-interaction (Tronick et al., 1979). The still face of the mother (an extreme case of incompatible mimicry) provokes clearly negative reactions in the infants, including withdrawal.

#### Marketing Communication

Understanding embodied resistance to persuasion may not only inform pro-consumer institutions but also advertising agencies and corporate units of market or consumer behavior analysis. For example, ad copy testing could include measures of behavioral expressiveness to check whether consumers engage in embodied resistance to persuasion. Integrated marketing communication could deliver customized experiences based on consumers’ embodied response profiles. That is, they should address consumers taking into account individual differences among them with regard to the non-verbal expression of emotions. In addition, the insights proposed based on our framework lead us to recommend salespersons acting in face-to-face situations to capitalize on naturally occurring mimicry responses of their audience. They should be able to influence purchase decisions of consumers by exhibiting facial expressions that when mimicked by the consumer, would increase their appetitive attitudes. Studies by van Baaren et al. (2003), Wang (2009) hint at such possibilities.

Our work is also relevant to marketers and advertisers who we know are using various “resistance-neutralizing tactics (…) tailored to the specific resistance strategy that is adopted by consumers” (Fransen et al., 2015b, p. 5). Marketers can benefit from understanding the mechanism behind the new consumer resistance strategy that we propose here.

## Limitations and Directions For Future Research

At least two exciting new avenues for the theoretical extensions of our framework open up thanks to the presentation of the basic premises of embodied resistance to persuasion in this paper. First, the consideration of the role of potential moderating factors in the causal paths of embodied resistance to persuasion is warranted. Below, in the limitations section, we identify as likely candidates motivation, persuasion knowledge, advertising skepticism, and emotion regulation as a trait. Second, to further develop the embodiment part of the framework, various modes of expression of emotion should be elaborated upon in more detail. That also means that the suppression of additional modes of expressing the emotion could have different, additive, multiplying, or no effects at all.

### Spontaneous Embodied Resistance to Persuasion

One significant limitation of our framework is that we ignore the role of consumers’ motivation to resist the ads. Some people may believe, for instance, that they are not affected by the ads, so they do not have to resist them. The existence of such a belief could be explained by the third-person effect (Davison, 1983), which argues that “a person exposed to a persuasive communication in the mass media sees this as having a greater effect on others than on himself or herself” (p. 1). Further, another issue related to the role of motivation to resist is people’s persuasion knowledge (Friestad and Wright, 1994) and their skepticism toward advertising (Obermiller and Spangenberg, 1998). Embodied resistance to persuasion could predominantly be a tool used by consumers with above average knowledge of persuasive communication strategies, or who are skeptical of advertising in general. Also environments that cue different types of motivation (e.g., telic or paratelic; see e.g., Lewinski, 2015d) could have different effects on the resistance.

Yet another concept that dovetails with consumers’ motivation to resist the ad in our model is emotion regulation as a stable trait. Emotion regulation is not only a temporal strategy moldable and sensitive to instruction, but also a chronic trait (Gross and John, 2003) measured through statements like, “When I want to feel more positive emotion (such as joy or amusement), I change what I’m thinking about” or “When I am feeling positive emotions, I am careful not to express them.” The evidence that is presented so far is restricted to strategies induced by instruction. For future studies it might be interesting to consider if spontaneous, and not instructed, emotion regulation stemming from, for example, individual differences would be equally effective in resisting advertisements.

Finally, an important question is left open. Under which conditions is an embodied resistance to persuasion strategy more effective than a “standard” strategy? Going even further, can we hypothesize that under some conditions, engaging in both kinds of resistance at the same time would offer the greatest benefit to the consumer? However, interesting as a possibility, this hypothesis remains to be supported by further research and conceptualizations.

### Other Forms of Expression of Emotion

In this paper, we did not consider the role of expression of emotion outside of facial expressions, though our framework allows for such possibilities. Alternative forms could encompass a head-down (sadness), clenched fist (anger), or straightened posture (pride). For example, sadness and anger are clearly negative emotions (Ekman et al., 1983) and should therefore decrease positive attitudes toward an entertaining advertisement. However, pride is an ambiguous emotion that is likely positive but also qualitatively different from, e.g., happiness and hence belonging to the same category as self-conscious emotions like embarrassment, shame, and guilt (Lewis, 2000). Thus, whether straightened posture would lead to lower or higher positive attitudes cannot be decided *a priori*. We did not consider those expressions because there is no automated way to code for them at any larger scale, a prerequisite for quantifying and interpreting significance and we could not have potentially generated enough empirical support to justify inclusion of those components explicitly in our model.

Further, e.g., Sweldens et al. (2010) showed that resistance instructions can counter explicit attitudes (and such were measured in the presented experimental data) but still influence implicit attitudes. This question is left open in our framework. It could be that emotion regulation, even though inhibiting facial expression would not further dampen implicit attitudes, i.e., attitudes captured by response time, IAT, galvanic skin response or other neuromarketing measures (such as fMRI or EEG), not filtered by any conscious verbalization process. However, because facial expression is itself a type of an implicit response in that sense, we predict that emotion regulation could be equally effective for resisting persuasion at an implicit attitude level. We think this is an exciting area to investigate in the future.

## Concluding Remarks

Commercials aiming to persuade are ubiquitous and making consumers aware of how to use their body in resisting these unwanted temptations could contribute to consumers’ empowerment. Embodied emotion regulation may be advocated as a novel and effective strategy to resist persuasion. To conclude, the working of embodied resistance to persuasion contributes to the growing scientific evidence that consumers’ bodily feedback has powerful regulatory effects on their behavior. In particular, our work highlights the role of bodily feedback in consumer resistance to persuasive ads. The downplaying of one’s emotional reactivity by either suppression or reappraisal empowers consumers, namely by helping them resist persuasive messages. We believe that embodied resistance to persuasion opens up a new avenue for persuasion research, showing that bodily emotion regulation mediated through facial behavior influences attitudes. In short, what we bodily express, influences not only how we feel and think but also whether or not we are persuaded.

## Author Contributions

PL, MF, and ET developed the outline, revised several drafts of the manuscript, prepared figures. PL performed the literature search, wrote all concept versions, presented preliminary results at research meetings and Etmaal 2015. MF and ET provided supervision.

## Conflict of Interest Statement

The authors declare that the research was conducted in the absence of any commercial or financial relationships that could be construed as a potential conflict of interest.
